# Age-related differences in postural adjustments during limb movement and motor imagery in young and older adults

**DOI:** 10.1007/s00221-020-05751-9

**Published:** 2020-02-27

**Authors:** Chloe Wider, Suvobrata Mitra, Mark Andrews, Hayley Boulton

**Affiliations:** Department of Psychology, Nottingham Trent University, Nottingham, UK

**Keywords:** Posture control, Motor imagery, Aging, Anticipation, Anticipatory

## Abstract

Recent research has shown that systematic postural adjustments occur during periods of manual motor imagery (MI), but the timing (anticipatory or reactive) and directionality (against or in the direction of arm extension) of these postural motions relative to individual manual actions or imagery are not well understood. This study analyzed the anteroposterior hip and head motion of healthy young and older participants, while they imagined bilateral arm raises under self-initiated or environmentally triggered performance conditions. When MI was self-initiated, both age groups showed significant forward postural motion during the second prior to MI initiation. When MI (or physical arm movement) was environmentally triggered, however, older people did not show anticipatory forward postural motion, but did show compensatory backward head motion. These results suggest that manual MI is indeed accompanied by anticipatory postural motion, but this anticipation is attenuated in older people when they do not have control over the timing of manual movement onset.

## Introduction

Goal-directed limb movement in upright humans is accompanied by concurrent postural control functions that prepare for and counteract the resulting perturbation to stance or gait (Massion 1992). Early studies on raising the arm while standing showed that leg muscles involved in postural control are the first to be activated (prior to the prime mover) (Belenkiy et al. 1967), and a backward bending of the trunk compensates for a forward motion of the center of gravity (CG) caused by forward arm movement (Martin 1967). Bouisset and Zattara’s work (1987a, b, 1988) on uni- and bilateral arm raising demonstrated that anticipatory postural adjustments (APA) act in the direction opposite to the reaction forces generated by arm movement, and Cordo and Nashner (1982) showed that forward body sway that would result from a handle pull is counteracted by anticipatory gastrocnemius muscle activity producing backward sway. The higher likelihood of observing APAs prior to fast (Lee et al. 1987) but not slow (Crenna et al. 1987; Horak et al. 1984, 1989) focal movements suggested that the purpose of APAs might be to protect the body’s balance from being disrupted by the perturbation caused by limb motion. Indeed, a key goal of APAs may be to regulate the CG (Bouisset and Zattara 1981, 1987b, 1988, 1990; Friedli et al. 1988; Ramos and Stark 1990) or its projection on the ground (Mouchnino et al. 1990; Rogers and Pai 1990).

More recently, Bleuse et al. (2006) observed that the counter-clockwise (viewed from above) vertical torque generated by raising the right arm from standing position is counteracted by an anticipatory clockwise torque. They suggested that this APA was produced to assist the arm movement by stabilizing the joints affected by it. The role of APAs in facilitating voluntary limb movements had also been suggested earlier by Lee et al. (1990) in the context of manual pulling movements. Based on the evidence that the duration of APAs increases with the load raised by the arm (Bouisset and Zattara 1988; Brown and Frank 1987; Zattara and Bouisset 1986), they suggested that APAs may provide additional force to focal movements, and, therefore, should be considered an integral aspect of voluntary movement control. In this respect, there is also evidence that APAs can contribute to movement initiation in the case of large forward movements of the body (Stapley et al. 1998).

The specificity of APA with respect to the associated focal movement suggests that the planning of both is functionally linked (Massion 1992). However, the adaptability of their relative timing also raises the possibility that APA production is a separate process from the control of focal limb movements (Brown and Frank 1987; Cordo and Nashner 1982). The close coordination between APAs and associated focal movements (e.g., APAs can be affected independently by the magnitude of perturbation and the magnitude of action triggering the perturbation) has suggested to some researchers that APAs should be considered integral aspects of focal movement planning (Aruin and Latash 1995, 1996). The finding that APAs can occur even when there is no focal movement (but a perturbation is predictable) suggests that APAs and corresponding focal movements are planned and controlled through two parallel processes of central origin (Aruin et al. 2001; Massion 1992).

The occurrence of postural adjustments in the absence of executed focal movement has also been studied in the context of motor imagery (MI) of limb movements (Boulton and Mitra 2013, 2015; Grangeon et al. 2011; Rodrigues et al. 2010). Boulton and Mitra (2013) showed that imagining goal-directed arm movements while standing upright elicits postural sway linked to task performance and that the control of postural sway during MI is of central origin (Boulton and Mitra 2015). Even though MI involves planning but not executing focal movement, it retains so many features of physical movements that it has been described as a simulation of physical action (Jeannerod 2006). MI exhibits scaling of movement time to distance (Decety et al. 1989; Papaxanthis et al. 2002; Sirigu et al. 1996), speed–accuracy tradeoff (Decety and Jeannerod 1995; Stevens 2005), the same adherence to biomechanical constraints (Frak et al. 2001; Johnson 2000), and the same pattern of simulated effort (Cerritelli et al. 2000) as its physical counterpart. MI also appears to share brain mechanisms for movement representation and execution (Bonnet et al. 1997; Clark et al. 2004; De Lange et al. 2006; Grèzes and Decety 2001; Orr et al. 2008), and has been found to generate corticospinal excitation (Stinear et al. 2006) and, in some situations, even specific but attenuated electromyographic (EMG) activity in involved muscles (Guillot et al. 2007; Lebon et al. 2008). As cerebral and corticospinal activation during MI can be modulated by modifying afference, for example, by immobilizing a limb (Kaneko et al. 2003), or enforcing an incompatible limb posture (De Lange et al. 2006; Vargas et al. 2004), it appears that the motor periphery is also referenced by the motor planning process involved in MI. These results suggest that MI involves detailed and specific motor planning (and even some preparatory aspects of motor execution), but no limb motion occurs, because an inhibition process of brain stem or spinal origin blocks the focal movement (Collet and Guillot 2009; Jeannerod 2006). If such inhibition exists, it must be incomplete in the sense that it does not block the autonomic arousal (Collet et al. 2013) or the postural adjustments that arise with motor planning (de Souza et al. 2015).

### The present study

Boulton and Mitra’s (2013, 2015) studies focused on demonstrating that MI was a cognitive task that could interfere with posture control because of the two tasks’ functional linkage resulting from the characteristics of MI noted earlier (Mitra et al. 2013; Stoffregen et al. 2007). As such, they focused on measuring postural sway during periods of imagined movements under specific MI and postural task conditions. This design allowed them to observe that MI-linked postural sway occurred, but it did not enable identification of the nature or direction of postural motion in the temporal vicinity of individual instances of imagined reaching movements of the arm. Grangeon et al. (2011) suggested that postural movement during MI could indicate unsuppressed APAs. Echoing the possible dual function of APAs outlined earlier, Boulton and Mitra (2013) considered both the possibility that their participants made APAs (and that these were larger when the imagined movements were expected to have a greater destabilizing effect on stance), and the possibility that postural motion was arranged to assist the reaching arm movements being imagined. The postural stabilization possibility was further supported when Mitra et al. (2016) found that older people restricted postural sway (even relative to quiet standing) where young people increased sway during MI of manual reaching movements. They interpreted this age-related reversal of response to MI as a postural threat response.

The literature on APA preceding physical arm movements has also found important age-related differences. APA preceding self-initiated body perturbations occurs later in older adults (Inglin and Woollacott 1988; Man’kovskii et al. 1980; Rogers et al. 1992), even when the velocities of the focal movements are not different between young and older adults (Woollacott and Manchester 1993). This delay in the onset of APA is thought to necessitate larger compensatory postural activity during focal movements in older adults (Kanekar and Aruin 2014a). Although most studies on age-related differences in APA onset have analyzed muscle activity, research has also shown that body motion associated with postural control (e.g., displacement of center of pressure and center of mass; Kanekar and Aruin 2014b) also occurs relatively later in older adults (Bleuse et al. 2006; Lee et al. 2015).

**Fig. 1:**
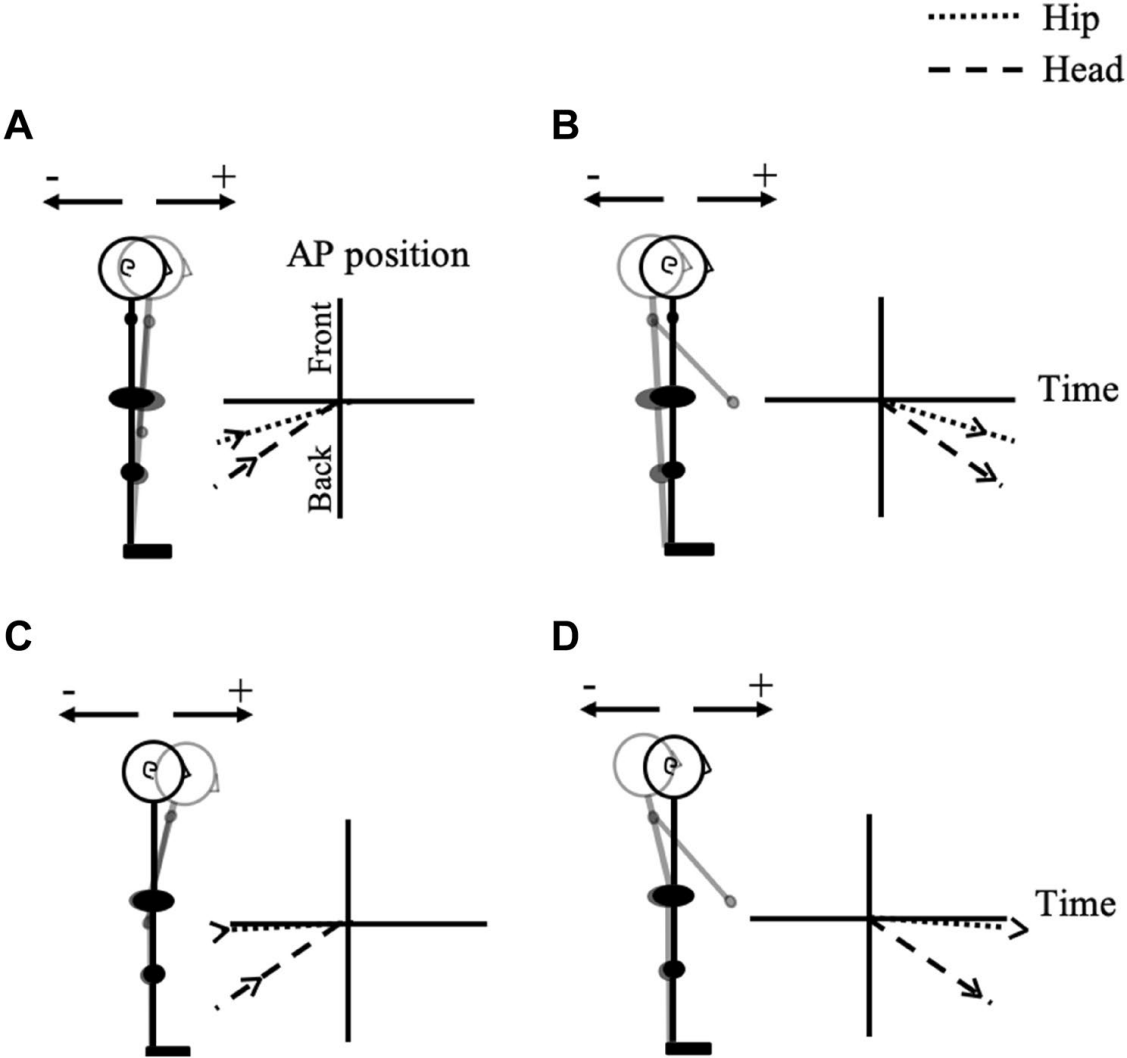
Schematic representation of anticipatory forward postural motion preceding the onset of arm extension (**a**, **c**) and compensatory backward postural motion during the arm’s extension (**b**, **d**). The vertical axis represents anteroposterior (AP) body position and the horizontal axis represents time. The origin is placed at the point in time which the arm movement is initiated, and the hip or head AP position at that time is assumed to have coordinate of zero. Panels **a** and **b** illustrate the expected motion in the case of postural adjustments made at the ankle joint (i.e., both the upper and lower body rotate), and panels **c** and **d** show the expected motion in the case of postural adjustment at the hip joint only (i.e., only the upper body rotates)

In the present research, we investigate the postural motion of standing young (Y) and older (O) participants’ head and hip in the 1000 ms preceding and following the onset of physical and imagined forward arm raising movements (Fig. 1). When postural motion seeks to minimize destabilization of the CG during the arm’s extension forward and up, we would expect to see either the backward motion of the upper and lower body (Fig. 1b), or the backward motion of the upper body only (Fig. 1d), corresponding, respectively, to the ankle and hip strategies (Nashner and McCollum 1985), or a mixture of the two. If this compensatory postural motion (CPM) moves the body backward, while the arm moves forward, any anticipatory postural motion (APM) preceding the onset of arm motion might be expected to take the body forward (Fig. 1a, c) (Bleuse et al. 2006; Bouisett and Zattara 1987a, b, 1988; Cordo and Nashner 1982). If this is the pattern we observe in the case of physical arm movement, an analogous forward motion of the body preceding imagined raising of the arm will point to APA accompanying MI. We limit ourselves here to a kinematic approach as the question of the nature of postural activity during MI arose in the context of kinematic studies (Boulton and Mitra 2013, 2015; Mitra et al. 2016). Informed by the kinematic patterns observed in this study, we consider the prospects of a surface EMG approach in the discussion.

We used the time window of 1000 ms preceding arm movement (or MI) onset to pick up the effects of both the early (preparatory) and the anticipatory postural activity that have been distinguished in the previous research (Krishnan et al. 2012; Lee et al. 1990). Post-arm movement (or MI) onset, a 1000 ms time window allowed the postural consequences of arm motion (or MI) to play out. In task conditions where the arm raise is imagined, a mechanical postural perturbation (as a result of the planned focal movement) does not in fact occur. Rodrigues et al. (2010) suggested that a mismatch between movement representations evoked by imagery and the subsequent absence of actual peripheral motor activity might have been responsible for the increase in postural sway they had observed in standing participants imagining plantar flexion movements. We predicted, therefore, that if MI elicits APM (i.e., forward body motion), reactive CPM should occur in the opposite (i.e., backward) direction in the post MI-onset period. Based on Mitra et al. (2016) finding of postural sway restriction in older adults during MI, we predicted that O would show reduced levels of postural motion relative to Y.

A final manipulation which we included was whether the physical or imagined arm movement was externally triggered (ET) by an experimenter-delivered ‘go’ signal or initiated by the participants at a time of their own choosing (self-initiated: SI). Boulton and Mitra’s (2013, 2015) and Mitra et al.’s (2016) experiments triggered physical and imagined reaching movements with an external signal. Their participants’ instructions were always to follow the ‘go’ signal immediately. The literature on APA preceding physical movements was initially thought to suggest that APA occurs only when a voluntary action generates a postural perturbation (Aruin and Latash 1995; Bennis et al. 1996; Dufosse et al. 1985; Johansson and Westling 1988; Massion 1992; Paulignan et al. 1989; Struppler et al. 1993), but Shiratori and Latash (2001) showed that APA can occur in the absence of voluntary limb motion when predictable perturbations are delivered externally. The issue of APA in the context of perturbations initiated by limb motion or an external perturbation is not the same as APA in the context of voluntary limb movements that start at a self-chosen time or are triggered by an environmental cue. However, initiating a movement in response to an expected environmental trigger, but with unpredictable timing, requires a motor plan to be held suspended until externally released, and the results of this process may differ from self-initiated action that does not require coordination with an unpredictable external trigger. To enable the detection of any age-related differences sensitive to this contrast, we carried out the present study under both ET and SI conditions.

## Methods

### Participants

Twenty young (8 male, 12 female; age range 18–29) and 20 older (6 male, 14 female; age range 65–88) individuals were recruited from the university and local communities, respectively, through the existing research participant panels. All participants reported no history of balance or neurological disorders and were right-handed according to the Edinburgh handedness inventory (Oldfield 1971). All had normal or corrected to normal vision. Each participant gave written informed consent and received a £10 retail voucher for coming to the session. Ethical approval for the research reported in this paper was granted by the Nottingham Trent University College of Business, Law and Social Sciences Research Ethics Committee.

All participants completed standardized tests of cognitive functioning. The Digit Symbol Substitution test from the Wechsler Adult Intelligence Scale-Revised (Wechsler 1981), with a maximum score of 94, was used to measure speed of information processing. The multiple-choice section of the Mill Hill vocabulary test (Raven et al. 1988), with a maximum score of 33, was used to measure vocabulary. Young (Y) and older (O) groups differed as expected, with significantly higher speed of information processing scores but lower vocabulary scores for Y than O (Salthouse 2010). The participant characteristics are summarized in Table 1.

**Table 1:** Participant characteristic means with SD in parentheses

	O	Y
Age (years)	72.85 (*6.15*)	23.65 (*3.51*)
Height (cm)	164.225 (*9.93*)	168.55 (*9.49*)
Weight (kg)	71.645 (*19.3*)	68.965 (*15.56*)
EHI	96.25 (*11.54*)	84.38 (*19.82*)
Mill Hill	22.45 (*3.93*)	17.8 (*4.44*)
DSS	50.1 (*7.92*)	68.2 (*11.08*)

Welch’s *t* tests showed that Y and O differed significantly in vocabulary (*t*(37.43) = − 3.51, *p* < 0.01) and speed of processing (*t*(34.40) = 5.94, *p* < 0.001)

*EHI* Edinburgh Handedness Inventory, *Mill Hill* vocabulary, *DSS* digit symbol substitution test of information processing speed (from WAIS-R)

### Apparatus

A four-sensor Codamotion motion-tracking system (Charnwood Dynamics, Rothley, UK) was used to record participants’ arm and postural motion. Active markers placed over the distal end of the middle metacarpal recorded arm motion. Markers located on the Codamotion pelvic frame placed horizontally over the posterior superior iliac spine recorded the hip’s postural motion and markers placed over the zygomatic bone recorded motion at the head. In addition, ground reaction force measurements were also taken, but are not reported here. The experimental protocol was controlled by an OpenSesame (Mathot et al. 2012) script that delivered instructions and the sequence of trials to participants and communicated with Codamotion’s Odin software to start and stop motion data acquisition.

### Procedure

For all trials, the participants were asked to stand barefoot in open stance (heels approximately 10 cm apart) and hold a computer mouse in their right hand. A computer monitor placed at eye level 2.5 m in front of the participant delivered the instructions for the experimental condition. Each trial started with a recorded voice saying “get ready”, which was followed by a random delay of up to 4000 ms. Following this, the recorded voice gave the “go” signal to make (or imagine) the arm raise. The instruction for the movement was to raise both arms to the front until they were parallel to the ground at shoulder level. Participants were asked to click the handheld mouse as they started and completed the arm movement.

In the self-initiated (SI) movement condition, participants were asked to wait at least 1000 ms after the “go” signal and then initiate (or imagine initiating) arm movement at a time of their own choosing. They were asked to make (or imagine) three SI movements, returning to the starting position after each. In the experimenter-triggered (ET) movement condition, participants moved (or imagined moving) their arm immediately upon hearing the “go” signal. As in the SI condition, movements were recorded in sets of three. For both the SI and ET conditions, only the first movement (or imagined movement) of each set of three was analyzed. This was because not all participants waited long enough in the SI condition between the first and second, or second and third movements, for the latter movements to be free of carryover effects.

The procedure for the MI trials was the same as for the physical movement trials, except that, instead of physically performing the movement, participants were asked close their eyes and imagine performing the same movement. They clicked the mouse when they started and ended the imagined movement, the same as when they performed the physical movements. The imagery instructions emphasized the kinesthetic element of MI by asking participants to imagine what it feels like to make the movement. The participants’ baseline sway pattern was recorded separately over a 60 s period during which they stood quietly with their eyes closed.

Physical movement and MI trials were blocked and block order was randomized. Experimental blocks consisted of five sets of three trials each (as described above). This blocked protocol allowed the participants to take frequent breaks and the experimenter to confirm data transfer from the Codamotion server to the data acquisition computer. In the physical movement conditions, participants completed three practice movements prior to performing the recorded ones. In the MI condition, participants initially completed three physical practice movements and then three imagined practice movements. This was to ensure that a physical movement was always completed before imagery if the imagery condition came first. This ensured a fresh memory of performing the physical movement when engaging in corresponding MI.

### Data analysis

**Fig. 2:**
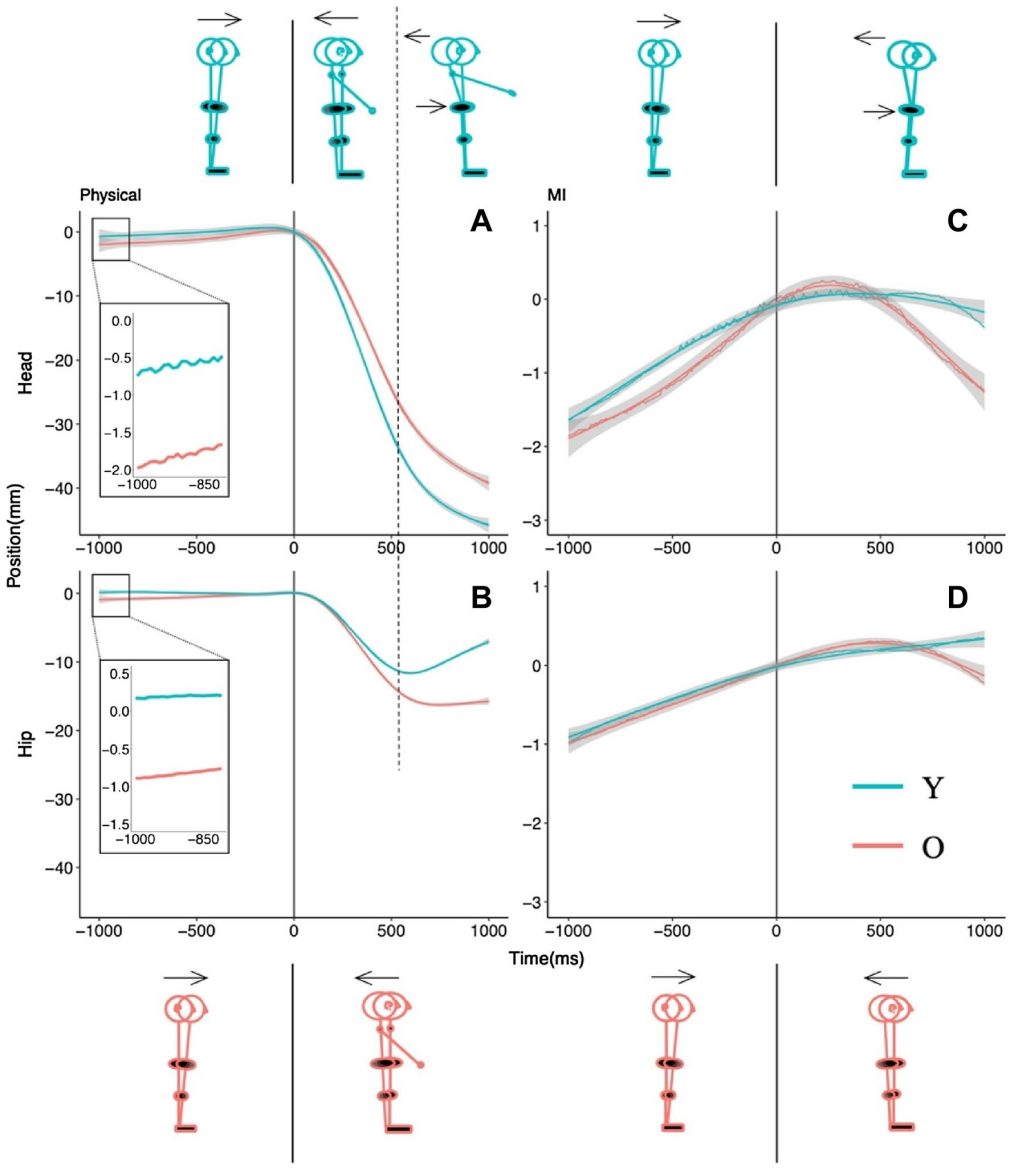
Postural motion in the vicinity of physical (**a**, **b**) and imagined (**c**, **d**) arm raising movements in the self-initiated (SI) condition. The upper panels show head motion and the bottom panels show hip motion

**Fig. 3:**
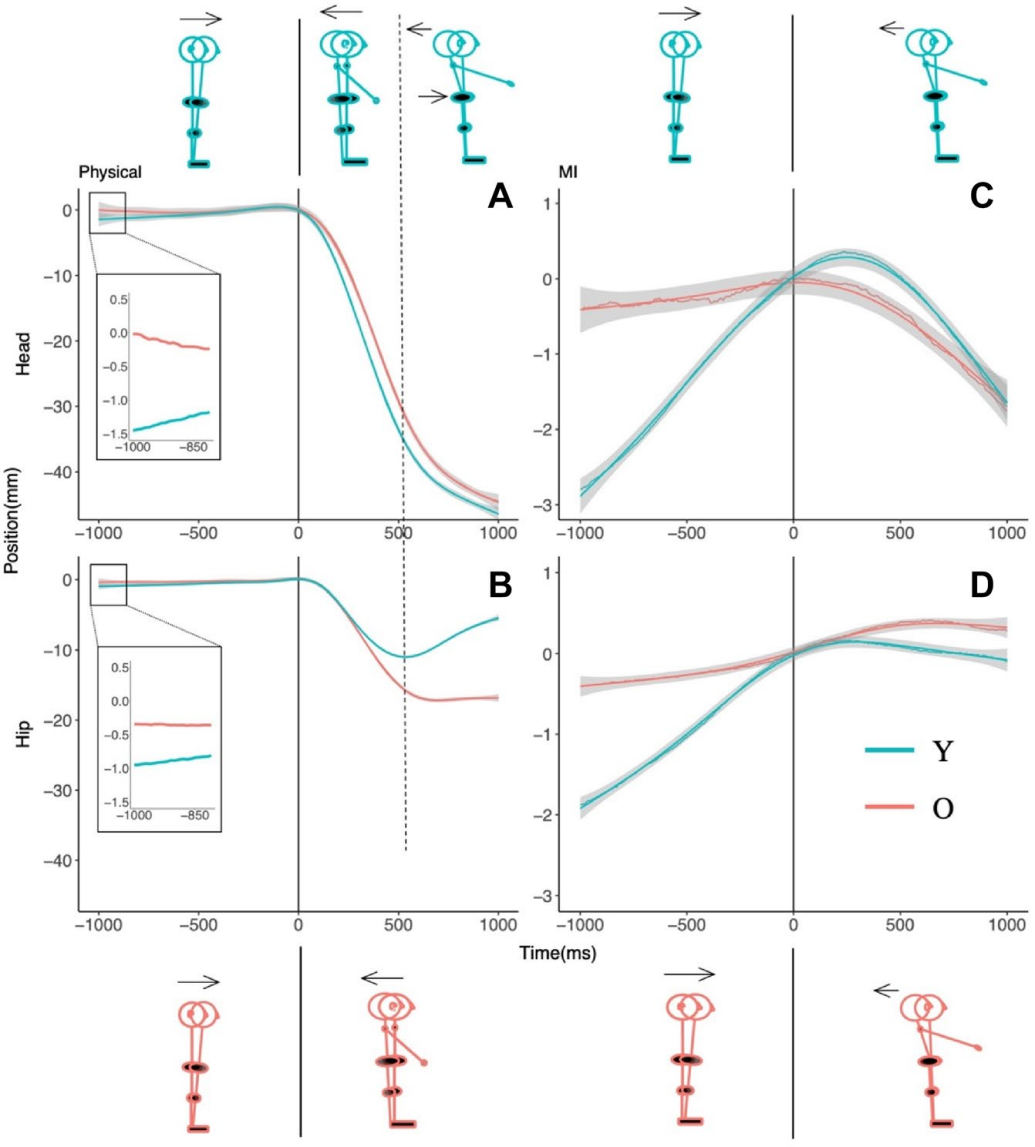
Postural motion in the vicinity of physical (**a**, **b**) and imagined (**c**, **d**) arm raising movements in the externally triggered (ET) condition. The upper panels show head motion and the bottom panels show hip motion

Data analysis focused on the anteroposterior (AP) postural motion of the hip and head segments, and the forward (horizontal) component of arm motion. In the physical movement conditions, the onset of arm motion was taken to occur when the forward velocity of the arm exceeded 1 m/s. Anticipatory postural activity was analyzed over the 1000 ms prior to this arm motion onset. Postural and arm motion was also tracked over 1000 ms following movement onset. In the case of MI trials, imagined movement was considered to have started when the participant indicated this by clicking the mouse button. For all recorded position time series, the time stamps of coordinate values were shifted, such that the point of arm movement initiation had *t* = 0 and AP position coordinate of zero (Figs. 1, 2, 3). This allowed all analyses of displacement and velocity data to be relative to the onset of arm motion (or imagined motion).

As shown in Figs. 2 and 3, participants’ APM in the physical and imagined arm movement conditions had approximately linear trajectories for both head and hip motion. In analyzing these cases, we took a multilevel linear modeling approach using lme4 (v1.06) in R (Bates et al. 2015; Magezi 2015). We fit Y and O’s hip or head position data to a varying slope and varying intercept model with time as a fixed effect and participants as a random effect. We termed this the test model. In this model, a positive slope (i.e., time coefficient) would indicate forward motion (as we expected for APMs). A zero slope in the body position trajectory before arm movement onset would signal the absence of APM. In this zero-slope case, the data would fit a baseline model that excluded the time coefficient of the test model. Thus, our first hypothesis test was to compare the test model against the baseline model for Y and O. If the test model fits the position data significantly better than the baseline model, we could conclude that there was significant forward (positive slope) postural motion during the time period in question. The next step was to take Y and O’s data together, and compare the test model to what we termed the theoretical model, the latter adding the participants’ age and the interaction between age and time to the test model. Our second hypothesis test was, therefore, to compare the theoretical model against the test model. If the theoretical model proved a better fit to the data (i.e., the time coefficient differed depending on age), we could conclude that Y and O exhibited different levels of APM.

In the case of CPM during physical arm movement and MI conditions (see Figs. 2, 3), the backward head and hip motions were not linear but curved. These CPMs had the shape of order 2 polynomials in the case of MI (Figs. 2, 3, right panels), and order 3 polynomials in the case of physical arm movement (Figs. 2, 3, left panels). In these cases, our analysis goal was simply to ascertain whether O and Y’s trajectories were statistically distinguishable. We did not seek to interpret the biomechanics underlying the trajectories in terms of the coefficients of our models. Our only interest beyond testing whether O and Y’s postural motion followed different paths after arm movement initiation was to note whether the phasing of head and hip motion during this time period differed between O and Y. We approached our analyses of CPMs in a manner analogous to our approach to analyzing APMs, except that we fit the third- and second-order polynomials in time in the case of physical arm movements and imagined arm movements, respectively. In both cases, the theoretical model was a varying intercept and slope model predicting AP position with age, time, time^2^ (and time^3^ in the physical arm movement case), and the interactions between age and each order of time as fixed effects, and participants as a random effect. This model was compared with a test model that excluded age and its interactions to test whether O and Y differed in their postural motion in this time period.

To compare any pair of linear mixed effects models, we performed a likelihood ratio test. Specifically, we calculated the difference in the log of likelihoods of the two models being compared. Under the null hypothesis that the two models are identical, − 2 times; this difference in the log of the likelihoods is distributed as a Chi-squared distribution with degrees of freedom equal to the difference in the number of parameters between the two models.

In addition to the aforementioned analyses, we also tested whether O and Y differed in their arm velocity profiles in the physical movement conditions. The purpose of this was to eliminate the possibility that any age-related postural differences which we observed were not due to differences in the speed with which O and Y raised their arm. For example, a comparative absence of (or reduction in) postural motion could be largely due to much slower arm motion in O. We also tested whether O and Y differed in their ability to refrain from moving their arm during the MI conditions. This was to ensure that any age-related postural differences observed in MI conditions could not be attributed to differences in uncontrolled arm motion. Finally, we also analyzed O and Y’s postural sway during quiet stance (i.e., in the absence of any manual or other task apart from standing upright) to eliminate the possibility that the types of postural motion which we observed and sought to interpret in the manual task conditions, especially the MI conditions, might also occur in the body’s natural sway in the absence of manual movement or MI.

## Results

In this section, we first present detailed results for anticipatory and compensatory postural motion observed during physical and imagined arm movements in the SI and ET arm movement conditions. At the end of the section, we provide a short summary of the main results.

### Self-initiated (SI) arm movement condition

Figure 2 summarizes the AP postural motion recorded just before and after arm movement (or MI) initiation in the SI condition. We first discuss the results for APM and then consider the case of CPM.

#### Anticipatory postural motion

AP sway recorded by the hip and head sensors in the 1000 ms preceding the onset of physical or imagined arm movement were analyzed separately. For each age group, we first ran the test model (to test whether there was a significant (linear) AP displacement prior to the onset of physical or imagined arm motion), and then compared it with the baseline model. Analogously, to test whether age affected the pattern of linear AP displacement in this time period, we compared the theoretical model to the test model.

The regression coefficients are shown in Table 2 and the postural motion trajectories in Fig. 2.

**Table 2:** Regression coefficients of the theoretical model for anticipatory and compensatory postural motion recorded at the head and hip segments in the self-initiated arm movement condition (see text for details)

Fixed effects	Anticipatory (self-initiated)			
	Head		Hip	
	Estimate	(SE)	Estimate	(SE)
Physical				
Intercept	− 1.01	0.65	− 0.47*	0.18
Time	0.69*	0.27	0.29**	0.10
Age (young)	0.96	0.92	0.54*	0.26
Time × age	− 0.27	0.38	− 0.38**	0.14
MI				
Intercept	− 1.08***	0.30	− 0.50**	0.17
Time	0.54**	0.18	0.28**	0.10
Age (young)	0.28	0.42	0.06	0.24
Time × age	0.06	0.26	− 0.01	0.14

Fixed effects	Compensatory (self-initiated)			
	Head		Hip	
	Estimate	(SE)	Estimate	(SE)
Physical				
Intercept	− 21.82***	1.79	− 10.58***	0.73
Time	− 848.31***	64.36	− 359.51***	31.19
Time^2^	114.10***	3.71	121.29***	2.42
Time^3^	97.02***	3.71	50.64***	2.42
Age (young)	− 5.36*	2.52	3.34**	1.03
Time × age	− 134.01	91.01	179.49***	44.11
Time^2^ × age	75.49***	5.26	34.46***	3.42
Time^3^ × age	7.55	5.26	− 14.98***	3.42
MI				
Intercept	− 0.24	0.38	0.16	0.24
Time	− 27.77	14.95	− 3.05	8.75
Time^2^	− 12.67***	0.57	− 8.10***	0.25
Age (young)	0.24	0.53	0.04	0.34
Time × age	24.25	21.14	7.88	12.38
Time^2^ × age	7.86***	0.81	8.12***	0.35

*, **, and ***, denote significance at the .05, .01, and ,001 levels, respectively

##### Physical arm movements

At the hip (Fig. 2b), O showed significant forward displacement of 0.98 mm (*χ*^2^(1) = 8.93, *p* < 0.01), but Y’s displacement of − 0.33 mm was not significantly different from zero (*χ*^2^ (1) = 0.88, *p* = 0.35). The difference between Y and O’s displacement was significant (*χ*^2^ (2) = 8.09, *p* = 0.02).

At the head (Fig. 2a), O had significant forward displacement of 2.35 mm (*χ*^2^ (1) = 6.66, *p* < 0.01), but Y’s displacement of 1.42 mm was not statistically distinguishable from zero (*χ*^2^ (1) = 2.02, *p* = 0.16). The difference between Y’s and O’s displacement was not significant (*χ*^2^ (2) = 1.71, *p* = 0.43).

Thus, O exhibited anticipatory forward motion at both the hip and the head but Y did not. O showed more forward motion than Y at the hip but not at the head.

##### Imagined arm movements

At the hip (Fig. 2d), both O (*χ*^2^ (1) = 6.23, *p* = 0.01) and Y (*χ*^2^ (1) = 7.75, *p* < 0.01) showed significant forward displacement of 0.93 mm and 0.97 mm, respectively, but the difference between Y and O’s displacement was not significant (*χ*^2^ (2) = 0.12, *p* = 0.94).

At the head (Fig. 2c), O (*χ*^2^ (1) = 5.02, *p* = 0.03) and Y (*χ*^2^ (1) = 12.34, *p* < 0.01) showed significant forward displacement of 1.85 mm and 1.66 mm, respectively, but the difference between Y and O’s displacement was not significant (*χ*^2^ (2) = 1.38, *p* = 0.50).

Thus, both O and Y exhibited significant anticipatory forward motion at both the hip and head, but there was no difference between the age groups.

#### Compensatory postural motion

As shown in Fig. 2, the compensatory postural motion trajectories were curved and were modeled as order 3 (physical arm movement, Fig. 2, left panels) or order 2 (imagined arm movement, Fig. 2, right panels) polynomials in time as previously described. The theoretical, test, and baseline models were established analogously to the procedure used for the analysis of APMs. The regression coefficients are shown in Table 2 and the postural motion trajectories in Fig. 2.

##### Physical arm movements

At the hip, the theoretical model showed that age, time, time^2^, time^3^, and all the interactions terms were significant predictors of AP position (Table 2 and Fig. 2b). When compared with the test model that excluded age and its interactions, the theoretical model provided a significantly better fit (*χ*^2^ (4) = 133.57, *p* < 0.01).

At the head, the theoretical model showed that age, time, time^2^, time^3^, and the interaction between age and time^2^ were significant predictors (Table 2, Fig. 2a). The theoretical model provided a significantly better fit to the data than the test model (*χ*^2^ (4) = 208.36, *p* < 0.01).

These results indicated that O and Y’s postural motion trajectories accompanying physical arm movement differed both at the head and the hip segments. The head showed a similar backward motion in O and Y (velocity was greater in Y), but the significant age × time^2^ interaction supports visual inspection in that O’s head velocity and displacement were lower than Y’s (Fig. 2a). Y’s hip motion was qualitatively different from Os; in that it showed forward motion following an initial backward motion. O’s hip motion did not show this recovery following initial backward motion (Fig. 2b). Given that the interactions between age and all three orders of time were significant in the test model, we conclude that Y initially had in-phase (backward) motion, but switched to anti-phase hip–head motion in the latter part of this time period. O’s hip motion plateaued following the initial in-phase backward motion, but did not reverse direction as for Y.

##### Imagined arm movements

At the hip, the theoretical model showed that time^2^ and the interaction between age and time^2^ were significant predictors of AP position (Table 2, Fig. 2d). When compared to the test model that excluded age and its interactions, the theoretical model provided a significantly better fit (*χ*^2^ (3) = 503.61, *p* < 0.01).

At the head also, the theoretical model showed that time^2^ and age × time^2^ were significant predictors of AP position (Table 2, Fig. 2c). Compared to the test model, the theoretical model fit significantly better (*χ*^2^ (3) = 96.66, *p* < 0.01).

These results suggested that O and Y followed parametrically different quadratic curves in their postural motion during imagined arm movement. Inspection of Fig. 2c, d shows that O’s AP motion reversed direction relative to the forward motion seen in the anticipatory phase. Y’s hip motion continued in the forward direction, albeit at a reduced rate, but Y’s head motion did reverse direction, although not as strongly as O’s.

### Environmentally triggered (ET) arm movement condition

Figure 3 summarizes the AP postural motion recorded before and after arm movement (or MI) initiation in the ET condition. We first discuss the results for APM and then consider the case of CPM.

#### Anticipatory postural motion

##### Physical arm movements

At the hip (Fig. 3b), O did not show significant forward displacement (0.37 mm) (*χ*^2^ (1) = 1.36, *p* = 0.24), but Y’s displacement of 0.87 mm was significantly different from zero (*χ*^2^ (1) = 10.16, *p* < 0.01). The difference between Y and O’s displacement was not significant (*χ*^2^ (2) = 1.65, *p* = 0.44).

At the head (Fig. 3a), O did not show significant forward displacement (0.51 mm) (*χ*^2^ (1) = 0.52, *p* = 0.50), but Y’s displacement of 2.02 mm was statistically distinguishable from zero (*χ*^2^ (1) = 5.94, *p* < 0.01). The difference between Y’s and O’s displacement was significant (*χ*^2^ (2) = 6.79, *p* = 0.03).

Thus, Y exhibited anticipatory forward motion at both the hip and the head, but O did not. Y showed more forward motion than O at the head but not at the hip.

##### Imagined arm movements

At the hip (Fig. 3d), O did not show significant forward displacement (0.35 mm) (*χ*^2^ (1) = 0.86, *p* = 0.35), but Y’s displacement of 1.99 mm was significantly different from zero (*χ*^2^ (1) = 17.48, *p* < 0.01). The difference between Y and O’s displacement was significant (*χ*^2^ (2) = 8.84, *p* = 0.01).

At the head (Fig. 3c), O did not show significant forward displacement (0.36 mm) (*χ*^2^ (1) = 0.24, *p* = 0.62), but Y’s displacement of 3.00 mm was statistically distinguishable from zero (*χ*^2^ (1) = 14.78, *p* < 0.01). The difference between Y’s and O’s displacement was significant (*χ*^2^ (2) = 8.00, *p* = 0.02).

Thus, Y but not O exhibited anticipatory forward motion at both the hip and head, and their difference was significant.

#### Compensatory postural motion

##### Physical arm movement

At the hip, the theoretical model showed that time, time^2^, time^3^, and the age × time^2^ and age × time^3^ interactions were significant predictors of AP displacement (Table 3 and Fig. 3b). When compared with the test model that excluded age and its interactions, the theoretical model provided a significantly better fit (*χ*^2^ (4) = 223.57, *p* < 0.01).

**Table 3:** Regression coefficients of the theoretical model for anticipatory and compensatory postural motion recorded at the head and hip segments in the externally triggered arm movement condition (see text for details)

Fixed effects	Anticipatory (externally triggered)			
	Head		Hip	
	Estimate	(SE)	Estimate	(SE)
Physical				
Intercept	− 0.14	0.48	− 0.25	0.19
Time	0.15	0.22	0.11	0.08
Age	− 0.44	0.68	− 0.31	0.27
Time × age	0.44	0.31	0.15	0.12
MI				
Intercept	− 0.25	0.41	− 0.25	0.12
Time	0.11	0.21	0.10	0.11
Age	− 1.16	0.58	− 0.74*	0.28
Time × age	0.77*	0.29	0.48***	0.16

Fixed effects	Compensatory (externally triggered)			
	Head		Hip	
	Estimate	(SE)	Estimate	(SE)
Physical				
Intercept	− 25.57***	1.78	− 11.51***	0.72
Time	− 976.06***	66.51	− 371.63***	24.28
Time^2^	157.32***	3.88	143.61***	2.62
Time^3^	120.68***	3.88	40.55***	2.62
Age (young)	− 2.83	2.52	4.70***	1.02
Time × age	3.62	94.06	253.17***	34.33
Time^2^ × age	72.59***	5.49	32.76***	3.7
Time^3^ × age	− 40.49***	5.49	− 39.51***	3.7
MI				
Intercept	− 0.61	0.52	0.29	0.22
Time	− 34.07	18.59	4.93	8.21
Time^2^	− 8.33***	0.70	− 4.83***	0.33
Age (young)	0.33	0.74	− 0.23	0.31
Time × age	− 2.37	26.28	− 8.74	11.61
Time^2^ × age	− 8.31***	0.99	3.06***	0.47

*, **, and ***, denote significance at the .05, .01, and ,001 levels, respectively

At the head as well, the theoretical model showed that time, time^2^, time^3^, and the age × time^2^ and age × time^3^ interactions were significant predictors of AP displacement (Table 3 and Fig. 3a). The theoretical model provided a significantly better fit to the data than the test model (*χ*^2^ (4) = 228.36, *p* < 0.01).

Thus, O and Y’s postural motion during physical arm movements differed both at the head and hip segments. The head’s backward motion was very similar in O and Y, but the interactions between age and the time^2^ and time^3^ terms express O’s lower head velocity and displacement (Fig. 3a). Y’s hip motion differed qualitatively from Os; in that it reversed its initially backward direction to recover. O’s backward hip motion had higher velocity but then plateaued rather than reverse direction like Y’s (Fig. 3b). Considering hip and head motion together, Y initially showed in-phase backward motion and then switched to anti-phase as hip position began moving forward. O initially showed in-phase hip and head motion but diverged when the hip’s backward motion stopped (without reversing direction).

##### Imaginary arm movement

At the hip, the theoretical model showed that time^2^ and the interaction between age and time^2^ were significant predictors of AP position (Table 3, Fig. 3d). When compared to the test model that excluded age and its interactions, the theoretical model provided a significantly better fit (*χ*^2^ (3) = 42.75, *p* < 0.01).

At the head also, the theoretical model showed that time^2^ and age × time^2^ were significant predictors of AP position (Table 3, Fig. 3c). Compared to the test model, the theoretical model fit significantly better (*χ*^2^ (3) = 71.30, *p* < 0.01).

These results indicated that O and Y followed different quadratic curves in their hip and head motion during imagined movement. Figure 3c, d shows that Y exhibited backward motion at both hip and head, but O showed backward motion only of the head.

### Arm movement peak velocity and its latency

**Fig. 4:**
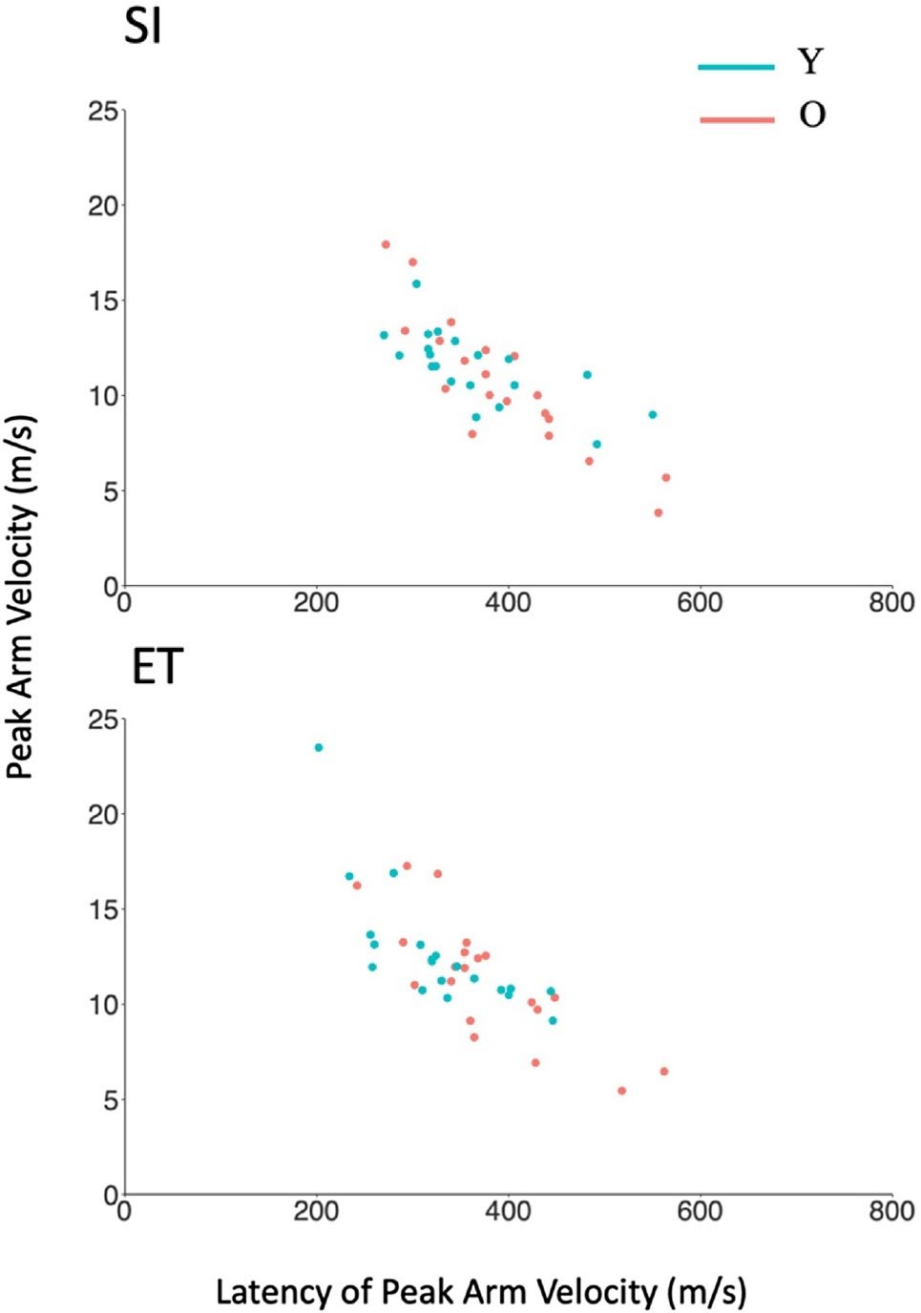
Peak arm velocity and its latency in the self-initiated (SI) and externally triggered (ET) conditions

For the experimental conditions in which the arm raise was physically performed, we investigated whether there were any age-related differences in the peak velocity attained by the arm and in the latency at which this occurred (Fig. 4). The theoretical model was a varying intercept and slope model predicting the right hand’s peak AP velocity and its latency with age and time as fixed effects and participant as a random effect. We compared this model with a test model that excluded the age coefficient.

In the SI condition, there was no difference between Y and O’s peak velocity (*χ*^2^ (1) = 1.01, *p* = 0.32) or its latency (*χ*^2^ (1) = 1.58, *p* = 0.21). In the ET condition, there was no difference between Y and O’s peak velocity (*χ*^2^ (1) = 1.75, *p* = 0.18), but O reached peak velocity later (374.00 ms, SD = 86.29) than Y (326.60 ms, SD = 73.95) (*χ*^2^ (1) = 4.29, *p* = 0.04).

### Arm motion during MI

In the experimental conditions in which the arm raise was to be imagined but not performed, we analyzed whether the arm exhibited any systematic forward or backward motion in the 1000 ms before or after the start of MI (indicated by participants’ mouse click). We used the same strategy as in the analysis of postural motion—the test model was a varying intercept and slope model predicting the right hand’s AP position with time as a fixed effect and participants as a random effect. This model was compared with a baseline model that excluded the time coefficient. We rejected the null hypothesis (no AP displacement in this time period) if the test model fit the data significantly better than the null model.

In the SI condition, O showed no significant arm motion in the pre-MI period (*χ*^2^ (1) = 0.93, *p* = 0.33), and Y showed marginally significant (*χ*^2^ (1) = 3.54, *p* = 0.06) forward motion. The magnitudes were 0.52 mm and 0.64 mm, respectively (compared to the 2.35 mm and 1.42 mm of head sway recording during this time period).

During the MI period, O showed marginally significant arm motion (*χ*^2^ (1) = 3.52, *p* = 0.06), but Y did not (*χ*^2^ (1) = 1.52, *p* = 0.22). Again, the magnitudes of 1.83 mm and 1.22 mm, respectively, were comparable to head motion recorded in this time period.

In the ET condition, O showed significant arm motion in the pre-MI period (*χ*^2^ (1) = 10.38, *p* < 0.01), and so did Y (*χ*^2^ (1) = 22.20, *p* < 0.01). The magnitudes were 1.25 mm and 2.02 mm, respectively, which were comparable to the 0.36 mm and 3.00 mm of head sway recorded during this time period.

During MI, O showed marginally significant arm motion (*χ*^2^ (1) = 3.70, *p* = 0.05), but Y did not (*χ*^2^ (1) = 0.42, *p* = 0.52). Again, the magnitudes of 3.17 mm and 0.52 mm, respectively, were comparable to head motion recorded in this time period.

These results show that arm motion was comparable or smaller than postural motion recorded from the upper body. We concluded, therefore, that both O and Y successfully inhibited focal arm movement during 1000 ms before and after self-reported MI onset.

### Postural sway in the quiet stance baseline condition

**Fig. 5:**
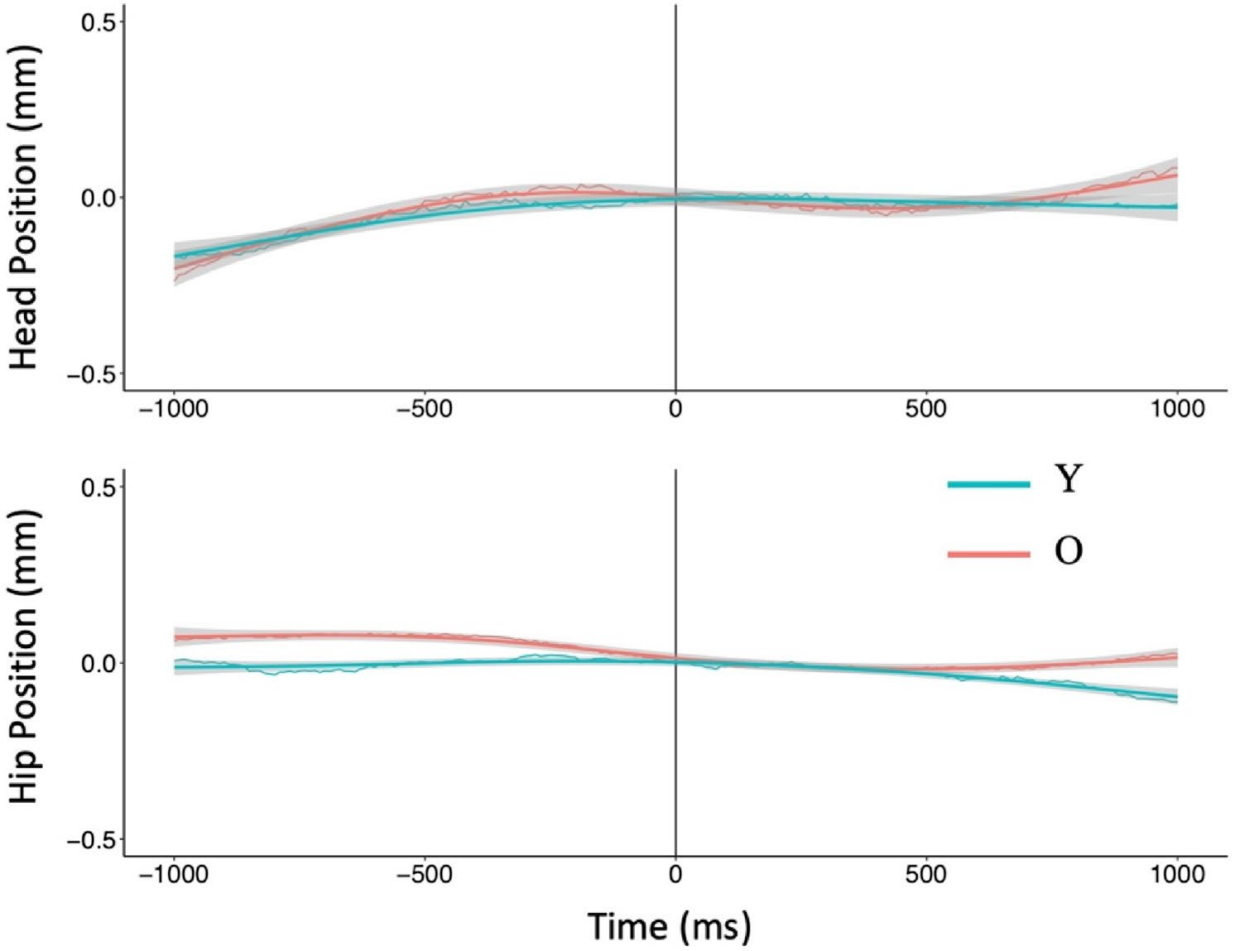
Average postural motion over 2000 ms time windows during 60 s quiet stance baseline condition

In the case of physical and imagined arm raises, we analyzed linear trends in AP postural sway over 1000 ms preceding and following the onset of arm motion (or MI). As we interpreted the observed linear trends as postural motion linked to the manual task, we also analyzed participants’ average sway over all 2000 ms periods during the 60 s quiet stance baseline condition to test whether any such linear trends occurred in the absence of manual task requirements. Figure 5 shows participants’ sway pattern during 1000 ms preceding and following the midpoint of the average 2000 ms time window during the baseline trial. It can be seen that sway relative to an arbitrary time point during quiet stance has much lower dispersion than was observed around the onset of physical and imagined arm movements in the experimental conditions. We performed the same statistical modelling on these data as in the experimental conditions and found no significant linear trends.

## Results summary

### Self-initiated arm movement

Anticipatory postural motion: Immediately before physically performing the arm movement, O showed significant forward displacement at the hip and head segments, while Y did not. Compared to Y, O showed more forward motion at the hip but not the head. Preceding imagined the arm movement, both age groups showed significant forward motion at the head and hip. O and Y’s head or hip motion did not differ from each other.

Compensatory postural motion: O and Y differed in their postural motion during physical and imagined arm movement at both segments. Y showed greater backward motion and greater reversal of this than O.

#### Environmentally triggered arm movement

Anticipatory postural motion: Preceding physical arm movements, O did not show forward displacement at the head or hip, whereas Y did. O and Y’s head motion, but not hip motion differed significantly. Preceding imagined arm movements, O did not show forward motion of the hip or the head, but Y did. The difference between the groups was significant at both the head and the hip.

Compensatory postural motion: Y and O’s hip and head motion followed statistically different quadratic curves during physical and imagined arm movements.

## Discussion

The purpose of this study was to investigate the spatiotemporal characteristics of the postural motion that accompanies physical and imagined arm movements in standing young and older adults. Y and O executed (or imagined) bilateral, straight-arm raises under SI or ET conditions. Y and O’s physical arm movements’ velocity profiles were very similar and differed only in O’s slower time to peak velocity in the ET condition. We consider CPMs and APMs observed around physical arm movement onset first, and then focus on postural motion observed in the context of manual MI.

In the case of physical arm movements, the forward displacement of the arms moves the body’s CG in the forward direction, so a backward CPM would be expected to stabilize the CG as the movement occurs (Bouisset and Zattara 1981, 1987b, 1988, 1990; Friedli et al. 1988; Mouchnino et al. 1990; Ramos and Stark 1990; Rogers and Pai 1990). Martin (1967) specifically observed that a backward bending of the trunk achieved this CG regulation. CPM analysis in this study showed that in both SI and ET conditions, Y and O had backward postural motion in the first 500 ms following arm movement initiation, Y with higher velocity of the hip and the head. In the next 500 ms, the behavior of Y and O diverged in a similar way in the SI and ET conditions. Y and O’s head motion continued in the negative direction (with decreasing velocity), but Y’s hip motion reversed direction to move forward, whereas O’s hip motion remained unchanged over this period. This pattern suggests that, as noted by Martin (1967), backward bending of the trunk was used to regulate CG as the arms extended forward. The inter-segmental phase change was simply more prominent in Y than in O.

If an APM precedes a forward movement of the arms, it ought to be in the forward direction, opposite to the backward CPM accompanying the movement (Bleuse et al. 2006; Cordo and Nashner 1982). In this study, analysis of APM preceding physical arm movement showed that O but not Y moved forward in the SI condition (Fig. 2a, b), whereas Y but not O did so in the ET condition (Fig. 3a, b). The differences between Y and O were subtle in the case of physical movements. In the SI condition, O showed more forward motion than Y at the hip but not at the head, and in the ET condition, Y showed more forward motion than O at the head but not at the hip. The forward direction of APMs (when they occurred) is consistent with expectation, but it is not clear what the age-related differences between the SI and ET conditions indicate.

In the SI condition, the perturbation due to arm motion was predictable, and given that the participants chose when to initiate the movement, so was the timing of movement onset. O’s forward APM was expected, but the absence of APM in Y was not. It could be that Y used a neuromuscular strategy such as co-contraction and so their anticipatory postural adjustment did not generate net forward motion. In the case of MI in the SI condition, both Y and O showed clear APM of similar magnitude in the forward direction. Y and O’s mean APM magnitudes (hip: 0.97 mm and 0.93 mm, and head: 1.66 mm and 1.85 mm, respectively) preceding MI were of the same order as O’s APM magnitude (hip: 0.98 mm, head: 2.35 mm) preceding physical movements. The MI data suggest that forward APM was planned by both Y and O.

For physical movements in the ET condition, Y’s APM in the forward direction was as expected, but O did not show statistically significant APMs. In this condition, it was predictable that arm movement would perturb posture control, but exactly when the go signal for the arm movement would arrive was not predictable due to the randomly variable latency between the ready and go signals. One possibility is that, under these conditions, O did not (or could not) plan and execute APMs. The APM data from the corresponding MI condition support this possibility as Y showed significant APM (hip: 1.99 mm, head: 3.00 mm), whereas O did not. As O did not show APM preceding physical arm movement or MI, it appears that the lack of control over arm movement (or MI) onset impeded O’s ability to prepare for the postural perturbation to come.

This leaves the pattern observed for postural motion following the onset of MI. As the planned arm motion does not in fact occur in the case of MI, any APM preceding MI onset would need to be compensated following MI onset to maintain balance. In the present case of imagined forward arm movement, the CPM would need to be in the backward direction, and this is generally what was observed for Y and O in both the SI and ET conditions. However, there were differences in hip–head phasing that are worth noting. In the SI condition (Fig. 2c, d), Y showed an anti-phase hip–head pattern (the head reversed to moving backwards while the hip continued forward motion), but O showed in-phase backward motion of hip and head. Comparing with the corresponding CPMs in the physical arm movement condition (Fig. 2a, b), both Y and O had the same CPM pattern in the MI condition as in the physical arm movement case. In the ET condition (Fig. 3c, d), Y and O showed a very small amount of hip motion, but their difference was significant; Fig. 3d suggests that Y had a more backward tendency at the hip that counteracted their forward APM prior to MI onset. Y’s backward CPM at the head counteracted their forward APM prior to MI onset. O showed the same pattern of backward head CPM as Y (Fig. 3c), but their head CPM followed next to no forward APM prior to MI onset. Thus, O ended the MI trials with a net backward head motion in the absence of forward arm motion, which will have been a destabilizing influence on their balance.

The present results clearly demonstrate that APM is a feature of motor behavior not only in the case of physical limb movement, as previous research has long established, but also, as raised by Boulton and Mitra (2013, 2015) and Grangeon et al. (2011), in the case of MI. In their comparison of the postural motion of Y and O, Mitra et al. (2016) found that sway increased in Y but decreased in O (relative to a quiet standing baseline), while they imagined reaching arm movements under ET conditions. Here, we observed that, unlike in the SI condition, O did not produce APM preceding MI in the ET condition. This absence of APM in the ET condition is consistent with the reduced sway recorded by O in Mitra et al. (2016). The reaching movements imagined in that study had more precisely defined targets, occurred only along the horizontal plane, and had smaller magnitudes than the bilateral arm raise studied here. Those task constraints may have added incentives for O to reduce body sway (e.g., to reduce shoulder motion to improve the precision of arm movement planning), but the absence of APM in O, which we observed here for both physical and imagined movements under ET conditions, appears likely to have contributed to O’s reduced sway during MI in Mitra et al. (2016).

The absence of APM preceding Os executed and imagined arm movement in the ET condition has potentially important practical consequences for active and independent living. Limb movements that must be coordinated with environmental events of unpredictable timing are an everyday necessity in navigating civic spaces and interacting socially. Raising the arm while standing upright does not even include the variable spatial constraints that are often added to the temporal uncertainties of coordinating with external events. Take, for example, the active destabilization of body posture that occurs when the trunk must bend as part of the focal movement, resulting in a large change in CG position (e.g., in Stapley et al. 1998). Previous research on postural support for physical movements has shown that O produces weaker and delayed APA (Inglin and Woollacott 1988; Man’kovskii et al. 1980; Rogers et al. 1992; Woollacott and Manchester 1993), and, as a result, larger CPA that can have destabilizing effects (Kanekar and Aruin (2014a). Here, O’s absence of APM for physical arm movements and MI in the ET condition suggests that the issue occurs at the level of planning the postural support for the movement that is to be coordinated with external events. Curiously, but potentially significantly, the absence of APMs coexists with intact CPMs even as no focal movement takes place.

This pattern raises questions for our understanding of the architecture of motor planning leading to physical or imagined limb movements. Massion (1992, Fig. 6a) summarized the control of focal movement execution and its postural support as parallel descending pathways of central origin. The assumption of separate pathways for controlling the focal and postural components was necessitated by the known flexibility of their relative timing depending upon task conditions (Benvenuti et al. 1990; Horak et al. 1984; Lee et al. 1987; Zattara and Bouisset 1986). On the evidence that the onset of focal movement can be held back until the required APA is fully developed (Cordo and Nashner 1982), an inhibition on the control of movement from the process that controls postural support was also postulated. Massion did not consider the case of MI, which involves a process that inhibits focal movement (Jeannerod 2006), and only recently, it has been demonstrated that postural adjustments (Boulton and Mitra 2013, 2015; Grangeon et al. 2011; Rodrigues et al. 2010) and autonomic preparation (Collet et al. 2013) planned in support of imagined movement can escape this inhibition. Massion also did not elaborate the architecture in respect of the anticipatory and compensatory components of posture control. The necessity of doing this is highlighted by the present observation of CPM in the absence of APM in O’s arm movement and MI in the ET condition.

Based on these considerations, we propose that the anticipatory and compensatory elements of the postural control pathway should be considered separable, such that the focal movement and compensatory postural support actions are tightly linked and co-occur in the case of movement execution. The anticipatory component may or may not occur depending upon its necessity and the ability to plan it. Where the whole process is externally triggered, for example, there may not be enough time or information to take anticipatory action. Also, previous and present results on movement execution, and present results on MI, suggest that old age brings with it a specific deficit in generating the anticipatory postural component when the focal movement’s timing must coordinate with an unpredictable external cue.

Throughout this report, we have been careful to distinguish APAs and CPAs, which have been studied in terms of patterns of postural muscle activation, and the APMs and CPMs that feature in our kinematic analysis. The presence of postural motion implies the presence of postural muscle activity to generate it, or the absence of muscle activity to resist it against gravity. The absence of postural motion, on the other hand, may signal either that no muscular effort was applied or that muscle activity occurred, but did not generate measurable body displacement (e.g., co-contraction of agonist–antagonist systems). Thus, further exploration of the ET task conditions combining kinematic and EMG measurement would be fruitful, although a surface EMG approach may be challenging if MI is associated with level-attenuated postural muscle activity. There seem to be at least two ways of amplifying the postural response accompanying MI. First, the focal limb movements could be made under added load, and second, a task condition could be introduced whereby postural contribution to the focal movement is necessitated. Also, the age-related deficit observed here could be probed more effectively by arranging the focal movement in the coronal plane, such that the postural adjustment required to counteract the reactive forces stress the particularly weakened mediolateral postural control in older people (Maki et al. 1994; Swannenburg et al. 2010).
